# The Role of Imaging in Diagnosing Transthyretin Cardiac Amyloidosis

**DOI:** 10.7759/cureus.15468

**Published:** 2021-06-05

**Authors:** Nourhan Chaaban, Shilpa Kshatriya

**Affiliations:** 1 Internal Medicine, University of Kansas School of Medicine-Wichita, Wichita, USA; 2 Cardiology, Heartland Cardiology, Wichita, USA; 3 Cardiology, University of Kansas School of Medicine-Wichita, Wichita, USA

**Keywords:** transthyretin, cardiac amyloidosis, hereditary attr amyloidosis, left ventricular hypertrophy, 99mtc-pyp scintigraphy, cardiovascular magnetic resonance imaging

## Abstract

Cardiac amyloidosis is a rare underdiagnosed condition with increasing morbidity and mortality. Its diagnosis is challenging and requires high clinical suspicion. Several diagnostic tools aid in the diagnosis of cardiac amyloidosis such as electrocardiogram, echocardiography, and, most importantly, cardiac MRI. A wide range of clinical symptoms is associated with cardiac amyloidosis, with shortness of breath and peripheral edema being the most common presenting complaints. Here, we report a case of transthyretin cardiac amyloidosis and discuss the importance of imaging in establishing the diagnosis.

## Introduction

Cardiac amyloidosis (CA) is a rare underdiagnosed condition with increasing morbidity and mortality. Transthyretin (ATTR) amyloidosis is currently considered the most frequent form of CA and its incidence is increasing thanks to the advances in diagnostic imaging techniques [1]. ATTR-cardiac amyloidosis (ATTR-CA) is classified into the hereditary form (hATTR) or non-hereditary form which is known as wild-type ATTR (wtATTR) based on the type of ATTR protein [2]. Non-hereditary ATTR-related amyloidosis is commonly referred to as senile systemic amyloidosis (SSA) because of its late age of onset (usually after the seventh decade of life). wtATTR has a strong male predominance, with between 25 and 50:1 male:female expression [3]. Prevalence is increasing with the progressively aging population and emerging diagnostic tools, including cardiovascular MRI and Technetium-99m-3,3-diphosphono-1,2-propanodicarboxylic acid (99m Tc-DPD) scintigraphy [4-5]. Therefore, we report a case of ATTR-CA that was initially suspected on imaging and was subsequently confirmed on biopsy.

## Case presentation

A 65-year-old white male with a previous history of hypertension was referred to the Heartland Clinic for evaluation of newly diagnosed pulmonary hypertension. He reported shortness of breath with exertion for one month in addition to leg swelling. His symptoms were so severe that he could no longer work. The patient denied palpitations, dizziness, or syncope. Family history was negative for cardiac disease including cardiomyopathy. A 2D echocardiogram showed markedly increased left ventricular wall thickness without left ventricular outflow tract obstruction, mild mitral regurgitation, mild left atrial dilatation, and mildly reduced left ventricular systolic dysfunction with an estimated ejection fraction of 45-50%.

On initial physical examination, blood pressure was 110/70 mmHg with heart rate (HR) of 77 beats per minute (bpm). There was jugular venous distention of 8 cm. Cardiac auscultation showed 2/6 left upper sternal murmur, normal S1S2, and regular rhythm. Lung auscultation was symmetrical, clear bilaterally. There was 2+ bilateral lower extremity edema with intact pulses. Electrocardiogram (ECG) showed normal sinus rhythm with left ventricular hypertrophy.

**Figure 1:**
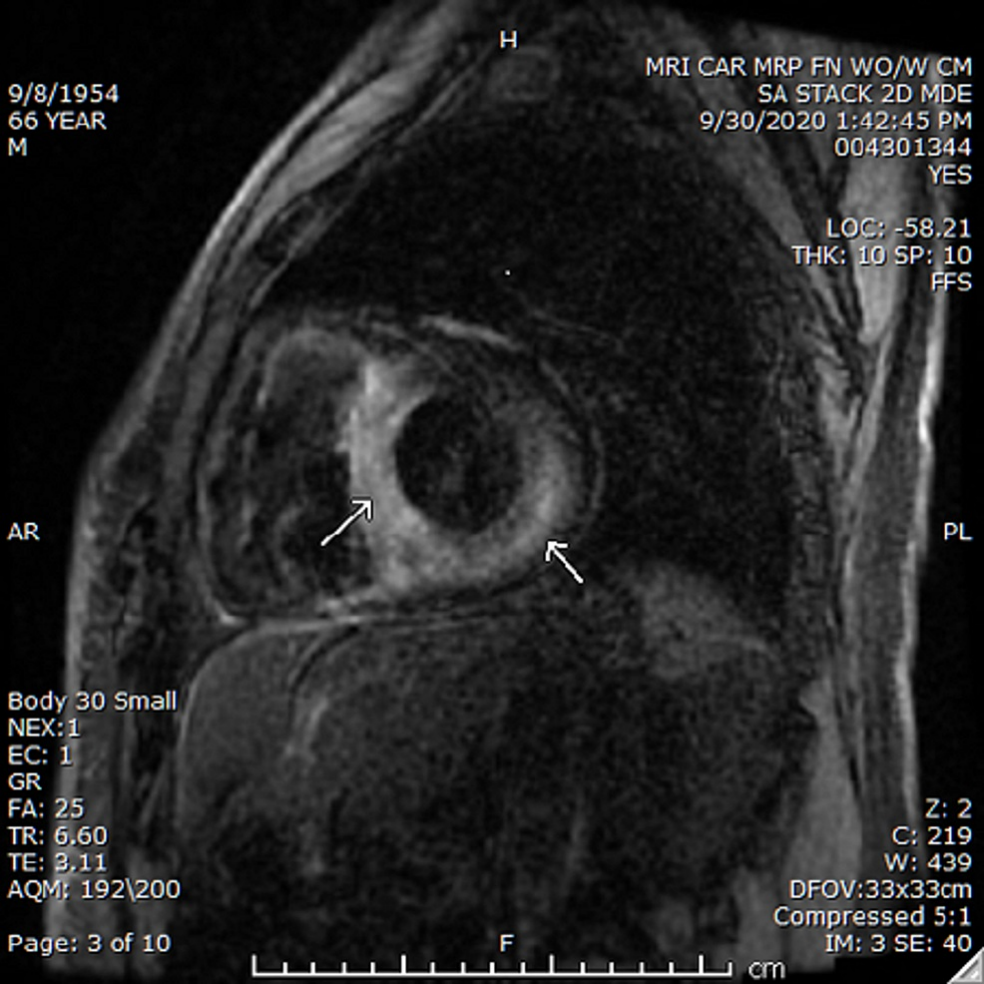
MRI of the heart with delayed gadolinium enhancement showing an enlarged heart with transmural delayed enhancement of the septal and lateral walls of the left ventricle; additional patchy subendocardial and mid-myocardial delayed enhancement is also seen involving the anterolateral wall.

Due to ongoing symptoms and high clinical suspicion of cardiac amyloidosis, contrast-enhanced cardiac MRI (Figure 1) was performed. It showed cardiomegaly with left ventricular hypertrophy present. There was a diffuse transmural enhancement in the septal, inferior, and lateral left ventricular walls along with enhancement of the lateral wall of the right ventricle. There was global hypokinesia of the left ventricle with a measured ejection fraction of 42% and trace pericardial fluid.

**Figure 2:**
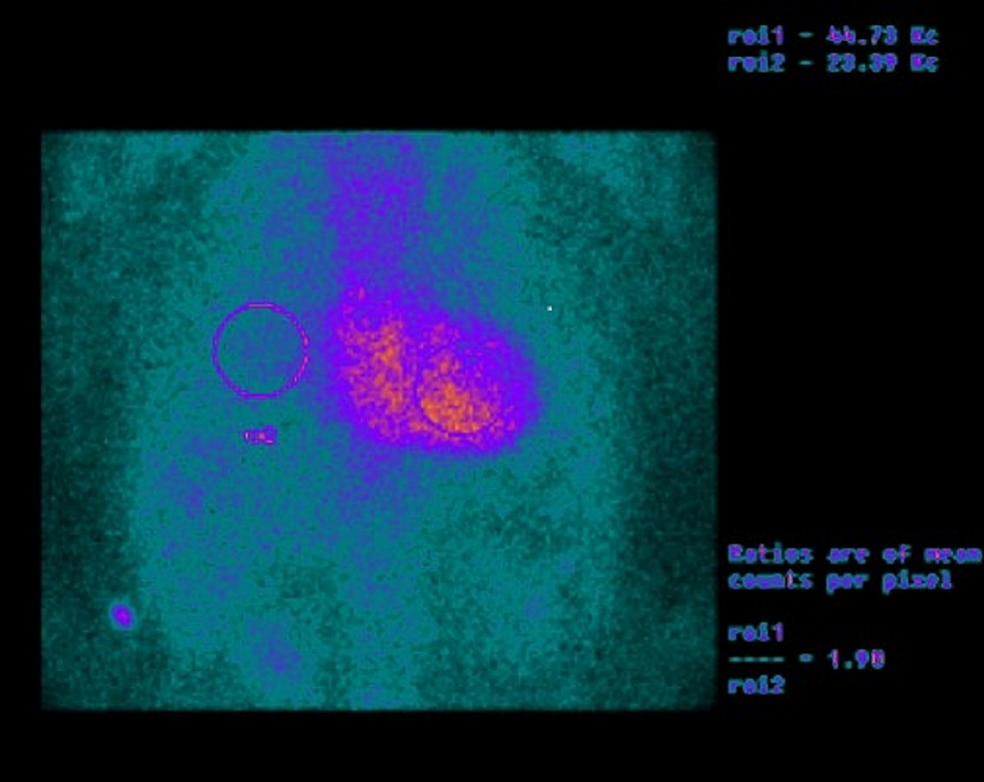
Nuclear imaging of 99mTc-PYP planar scintigraphy showing significant uptake of cardiac 99m Tc-PYP in the myocardium compared to the ribs. Circular target ROI are drawn over the heart on the planar images as well over the contralateral chest. A H/CL ratio is calculated as the fraction of heart ROI mean counts to contralateral chest mean counts. H/CL ratios of >1.5 are classified as ATTR positive at one hour post-injection. 99mTc-PYP: 99mTechnetium pyrophosphate; ATTR: Transthyretin; H/CL ratio: Heart-to-contralateral ratio; ROI: Regions of interest.

Nuclear imaging of 99mTechnetium pyrophosphate (99mTc-PYP) planar scintigraphy was ordered. It showed intense radiotracer uptake by the heart that was greater than normal bone uptake (visual score of Grade 3) and a quantitative heart to the contralateral ratio of 1.9 (>1.5 considered positive); both strongly suggestive of ATTR amyloidosis (Figure 2). Serum and urine protein electrophoresis was performed and were unremarkable.

A right heart catheterization revealed severe pulmonary hypertension with mean pulmonary artery pressure of 40 mmHg, pulmonary capillary wedge pressure (PCWP) of ­­­­26 mmHg, and pulmonary vascular resistance (PVR) of 1.85 suggestive of elevated left-sided filling pressures and cardiac etiology of pulmonary hypertension (post-capillary). Subsequently, a right ventricle biopsy was performed, and six specimens were taken from the endomyocardium. Microscopic examination showed perimyocytic and nodular deposits of eosinophilic amorphous material in several of the fragments. This material stained positive for amyloid on thioflavin S stain examined under fluorescence microscopy. Immunohistochemical staining was performed for amyloid typing. ATTR immunostaining was positive in the amyloid deposits. Lambda and kappa light chains were negative. The histologic and immunostaining patterns were consistent with the ATTR type of amyloidosis.

The patient was started on tafamidis 61 mg capsule daily. He has regular clinic follow-ups since diagnosis and has noted improved symptoms of shortness of breath and edema.

## Discussion

Amyloidosis is a rare multisystemic disease caused by the deposition of misfolded proteins in various organs such as the kidney, liver, heart, and nervous system. Many types of amyloidosis exist, differentiated by the nature of the deposited proteins, however, the light chain amyloidosis (AL) and ATTR amyloidosis proteins represent the majority of CA [6].

Most commonly, electrocardiography in CA shows left ventricular hypertrophy (LVH). Unexplained left LVH, characterized by low QRS voltage on electrocardiogram despite LVH on echocardiogram, provides valuable clues for the suspicion of CA. However, these electrocardiographic features are actually not sensitive enough to identify CA [7-8]. Less common ECG abnormalities can be present such as repolarization abnormalities, ischemic and T-wave inversions, and arrhythmic abnormalities such as atrial fibrillation. In our case, EKG showed normal sinus rhythm (NSR) with LVH.

Echocardiography is the modality of the first approach in the diagnosis of CA due to easy accessibility. The amyloid phenotype is typically characterized by biventricular wall thickening with small, non-dilated ventricles and left ventricular (LV) wall thickness, typically greater than 12 mm [9]. However, this finding is non-specific since it overlaps with other etiologies such as hypertension, aortic stenosis, hypertrophic cardiomyopathy, and other infiltrative cardiac diseases. Well-described but non-specific findings of CA include a thickened and sparkling appearance of the valves and interatrial septum, as well as a ‘speckled’ appearance of the myocardium [10]. These findings are due to the deposition of amyloid proteins which are more echogenic than the surrounding myocardial tissue. Compounding matters is that most of these features are not present until late in the diagnosis. 

Cardiovascular magnetic resonance (CMR) has established a significant role in diagnosing CA when suspected. CMR is especially useful in patients with increased left ventricular wall thickness and/or hypertrophy because it can differentiate different cellular and interstitial pathologies, which is not possible on echocardiography [11]. In CA, the amyloid fibrils deposit in the extracellular matrix leading to the expansion of the myocardial extracellular volume. Hence, through the administration of the gadolinium-based contrast agents, referred to as late gadolinium enhancement (LGE), one can visualize the accumulation of this agent between the myocardial cells and can differentiate between normal and abnormal myocardial tissue. Tissue characterization specifically with LGE imaging, including transmural LGE, large diffuse annular LGE, global heterogeneous LGE of “patch” LGE, has been reported to be one of the most accurate predictors of endomyocardial biopsy-positive amyloidosis [12]. However, this feature is not pathognomonic of CA as it might be visualized in other infiltrative diseases. Also, an important limitation of the application of contrast-enhanced CMR in TTR CA is co-existent chronic kidney disease and the risk of nephrogenic systemic fibrosis (NSF) [13].

Nuclear imaging is a sensitive tool for the identification of the TTR subtype of CA, and it is encouraging in distinguishing between it and the AL subtype which can also involve the heart. There are many radiotracers, and the most common single-photon emission CT tracer is a 99m Technetium-phosphate derivative. The exact mechanism behind the effectiveness of this imaging modality remains somewhat elusive, though there are a few plausible theories. Of these, it suggests that phosphate in the radiotracers binds to high calcium levels in amyloidosis. Another hypothesis is based on the duration of amyloid deposition that occurs in less time frame in patients with AL relative to a more indolent course of patients with ATTR. As 99mTc-PYP preferentially binds to ATTR relative to AL fibrils, this technique can also be used to distinguish the aforementioned amyloidosis subtypes [14].

## Conclusions

This case demonstrates the advanced utility of current imaging modalities to better diagnose CA so that treatment options can be introduced at an earlier stage. In the future, these advanced imaging modalities may assist with avoiding invasive procedures such as endomyocardial biopsy which is considered the gold standard. In the end, the early detection of cardiac amyloidosis is critical since treatment options are currently available with the goal of improving the quality of life and survival in affected patients. Current research has shown promise that the aforementioned imaging modalities may also help assess response to therapy.

## References

[1] Povar Echeverría M, Auquilla Clavijo PE, Escobedo Palau JA, Navarro Beltrán P, Povar Marco J (2018). Non-invasive diagnosis of cardiac amyloidosis due to transthyretin. Case report. An Sist Sanit Navar.

[2] Sipe JD, Benson MD, Buxbaum JN, Ikeda S-I, Merlini G, Saraiva MJ, Westermark P (2016). Amyloid fibril proteins and amyloidosis: chemical identification and clinical classification International Society of Amyloidosis 2016 Nomenclature Guidelines. Amyloid.

[3] Ruberg FL, Berk JL (2012). Transthyretin (TTR) cardiac amyloidosis. Circulation.

[4] Fontana M, Banypersad SM, Treibel TA (2014). Native T1 mapping in transthyretin amyloidosis. JACC Cardiovasc Imaging.

[5] Gillmore JD, Maurer MS, Falk RH (2016). Nonbiopsy diagnosis of cardiac transthyretin amyloidosis. Circulation.

[6] Perugini E, Guidalotti PL, Salvi F (2005). Noninvasive etiologic diagnosis of cardiac amyloidosis using 99mTc-3,3-diphosphono-1,2-propanodicarboxylic acid scintigraphy. J Am Coll Cardiol.

[7] Nijst P, Tang WW (2021). Recent advances in the diagnosis and management of amyloid cardiomyopathy. Fac Rev.

[8] Cyrille NB, Goldsmith J, Alvarez J, Maurer MS (2014). Prevalence and prognostic significance of low QRS voltage among the three main types of cardiac amyloidosis. Am J Cardiol.

[9] Sperry BW, Vranian MN, Hachamovitch R, Joshi H, McCarthy M, Ikram A, Hanna M (2016). Are classic predictors of voltage valid in cardiac amyloidosis? A contemporary analysis of electrocardiographic findings. Int J Cardiol.

[10] Lachmann HJ, Hawkins PN (2006). Systemic amyloidosis. Curr Opin Pharmacol.

[11] Chacko L, Martone R, Cappelli F, Fontana M (2019). Cardiac amyloidosis: updates in imaging. Curr Cardiol Rep.

[12] Wechalekar AD, Schonland SO, Kastritis E (2013). A European collaborative study of treatment outcomes in 346 patients with cardiac stage III AL amyloidosis. Blood.

[13] Vogelsberg H, Mahrholdt H, Deluigi CC (2008). Cardiovascular magnetic resonance in clinically suspected cardiac amyloidosis: noninvasive imaging compared to endomyocardial biopsy. J Am Coll Cardiol.

[14] Zou Z, Zhang HL, Roditi GH, Leiner T, Kucharczyk W, Prince MR (2011). Nephrogenic systemic fibrosis: review of 370 biopsy-confirmed cases. JACC Cardiovasc Imaging.

